# A community-based healthcare package combining testing and prevention tools, including pre-exposure prophylaxis (PrEP), immediate HIV treatment, management of hepatitis B virus, and sexual and reproductive health (SRH), targeting female sex workers (FSWs) in Côte d’Ivoire: the ANRS 12381 PRINCESSE project

**DOI:** 10.1186/s12889-021-12235-0

**Published:** 2021-12-04

**Authors:** Valentine Becquet, Marcellin Nouaman, Mélanie Plazy, Aline Agoua, Clémence Zébago, Hervé Dao, Alice Montoyo, Aude Jary, Patrick A. Coffie, Serge Eholié, Joseph Larmarange, and the ANRS 12381 PRINCESSE team

**Affiliations:** 1 Ined, Aubervilliers, France; 2 Ceped, IRD, Université de Paris, Inserm, Paris, France; 3 PAC-CI, Abidjan, Côte d’Ivoire; 4 Département de Santé Publique, UFR d’Odonto-Stomatologie, Université Félix Houphouet-Boigny, Abidjan, Côte d’Ivoire; 5 Bordeaux Population Health Research Center, Université de Bordeaux, Inserm, IRD, Bordeaux, France; 6 ONG Aprosam, San Pedro, Côte d’Ivoire; 7 ANRS, Paris, France; 8 Sorbonne Université, INSERM, Institut Pierre Louis d’Epidémiologie et de Santé Publique (iPLESP), AP-HP, Hôpital Pitié-Salpêtrière, Service de Virologie, Paris, France; 9 Département de Dermatologie et Infectiologie, UFR des Sciences Médicales, Université Félix Houphouet-Boigny, Abidjan, Côte d’Ivoire

**Keywords:** HIV prevention, Sexual and reproductive health, Sexually transmitted infections (STIs), hepatitis B, Pre-exposure prophylaxis (PrEP), Sex work, Mixed-methods research, Mobile clinics, Côte d’Ivoire

## Abstract

**Background:**

Pre-exposure prophylaxis (PrEP) is recommended by the WHO for HIV prevention among female sex workers (FSWs). A study conducted in 2016–2017 in Côte d’Ivoire showed that if PrEP is acceptable, FSWs also have many uncovered sexual health needs. Based on this evidence, the ANRS 12381 PRINCESSE project was developed in collaboration with a community-based organization. The main objective is to develop, document, and analyze a comprehensive sexual and reproductive healthcare package among FSWs in Côte d’Ivoire.

**Methods:**

PRINCESSE is an open, single-arm interventional cohort of 500 FSWs in San Pedro (Côte d’Ivoire) and its surroundings. Recruitment started on November 26th, 2019 and is ongoing; the cohort is planned to last at least 30 months. The healthcare package (including HIV, hepatitis B, and sexually transmitted infection management, pregnancy screening, and contraception) is available both at mobile clinics organized for a quarterly follow-up (10 intervention sites, each site being visited every two weeks) and at a fixed clinic.

Four waves of data collection were implemented: (i) clinical and safety data; (ii) socio-behavioral questionnaires; (iii) biological data; and (iv) in-depth interviews with female participants. Four additional waves of data collection are scheduled outside the cohort itself: (i) the medical and activity records of Aprosam for the PRINCESSE participants; (ii) the medical records of HIV+ FSW patients not participating in the PRINCESSE cohort, and routinely examined by Aprosam; (iii) in-depth interviews with key informants in the FSW community; and (iv) in-depth interviews with PRINCESSE follow-up actors.

**Discussion:**

The PRINCESSE project is one of the first interventions offering HIV oral PrEP as part of a more global sexual healthcare package targeting both HIV- and HIV+ women. Second, STIs and viral hepatitis B care were offered to all participants, regardless of their willingness to use PrEP. Another innovation is the implementation of mobile clinics for chronic/quarterly care. In terms of research, PRINCESSE is a comprehensive, interdisciplinary project combining clinical, biological, epidemiological, and social specific objectives and outcomes to document the operational challenges of a multidisease program in real-life conditions.

**Trial registration:**

The PRINCESSE project was registered on the Clinicaltrial.gov website (NCT03985085) on June 13, 2019.

**Supplementary Information:**

The online version contains supplementary material available at 10.1186/s12889-021-12235-0.

## Background

Most countries in West Africa have mixed HIV epidemics: a limited HIV prevalence in the general population and some key populations that are overwhelmingly affected, particularly female sex workers (FSWs) and men who have sex with men (MSM) [1]. In 2019, 19% of new HIV infections in West and Central Africa occurred among FSWs and 27% among clients of FSWs and other sexual partners of key populations [1]. In Côte d’Ivoire, HIV prevalence among FSWs was estimated to be 11.4% in Abidjan in 2014 [2], and HIV incidence was 3.2% in San Pedro and 1.5% in Abidjan in 2016–2017 [3] vs. 0.5% in the general population in 2019 [4]. FSWs are exposed to HIV, as they do not systematically use condoms with their male partners—primarily because of coercion, the primacy of men’s sexual pleasure, or to obtain protection from their partner against the threat of violence [5–7] —or with their clients in order to earn more, or because of violence from some clients [5, 8–12]. However, this population often lacks access to adequate services to prevent HIV acquisition and access to HIV care when HIV-positive [13].

In this global context, a new tool for HIV prevention was developed in the last decade: oral pre-exposure prophylaxis (PrEP), which consists of antiretroviral drugs taken by HIV-negative people to prevent HIV acquisition. When taken properly, oral PrEP has been shown to be very effective in preventing HIV acquisition, with a relative reduction of 75 to 86% [14–16]. Since 2015, oral PrEP has been recommended by the WHO for populations “at substantial risk” of being infected by HIV [17], such as FSWs. However, the effective implementation of PrEP raises questions, particularly among women, and needs more operational research [17].

First, clinical trials conducted in South and East Africa have shown low adherence among women, resulting in little or no effect of PrEP on HIV acquisition [18, 19]. Likewise, implementation trials conducted in Africa among FSWs have revealed relatively low retention, even when a priori acceptability of PrEP was high [20–22]. Second, PrEP does not protect against sexually transmitted infections (STIs) or unwanted pregnancies; sexual and reproductive health (SRH) needs beyond HIV must therefore be addressed [23]. More specifically, in Côte d’Ivoire, oral PrEP is not yet implemented at scale, and the National AIDS Programme has been asking for operational research before scaling up.

In 2016–2017, we conducted the ANRS 12361 PrEP-CI, a cross-sectional and mixed-methods study, to explore sexual healthcare needs that should be considered within a PrEP program targeting FSWs in Côte d’Ivoire, in order to better describe the experiences of FSWs who are reached via peer educators, and to test the pertinence and a priori feasibility of such programs [5]. Implemented at prostitution sites in two Ivorian cities (Abidjan and San Pedro) in collaboration with two Ivorian community-based organizations (Espace Confiance and Aprosam), the PREP-CI study included (i) a quantitative survey among 1000 FSWs who had never been tested or previously tested HIV-negative, including a socio-behavioral questionnaire, HIV testing, and, for those who tested HIV-positive, collecting a dried blood spot for a recent infection assay to estimate HIV incidence; and (ii) a qualitative survey based on individual interviews and focus-group discussions among 66 FSWs. A final workshop was organized with six community non-governmental organizations (NGOs) and the National AIDS Programme to discuss the main results and to elaborate on an operational research project. Thus, the ANRS 12381 PRINCESSE project was developed based on evidence generated by this PrEP-CI study.

### All FSWs have unmet SRH needs, whether they are infected with HIV or wish to initiate PrEP

The community clinics dedicated to FSWs are mainly frequented by HIV+ FSWs, despite the existence of services for all FSWs. However, their health needs go beyond HIV prevention and care. The non-systematic use of condoms exposes FSWs to STIs and unwanted pregnancies, which increase morbidity and mortality [24]. According to two studies conducted in Côte d’Ivoire in 2014, 70% of FSWs who have ever been pregnant have had at least one abortion [2, 25], the majority of them clandestinely, as abortion is illegal in Côte d’Ivoire (except for saving the mother’s life or in the case of rape since 2019). In the PrEP-CI study, 50% of the surveyed FSWs said they had had at least one abortion in their lifetime, and 65% reported having contracted an STI in the past 12 months. Qualitative interviews revealed the frequent use of cloth or cotton pieces for menstrual hygiene, a source of bacterial infection [26, 27].

Further, contraceptive prevalence is low, despite a high risk of unintended pregnancy. In the PrEP-CI study, 42% of FSWs declared having had an unwanted pregnancy, but 61% were not using contraception other than condoms, thus confirming trends already observed in Abidjan in 2014 [2, 25]. The interviews revealed that FSWs did not use modern contraception for fear of becoming infertile. Hence, there is a need to enhance their sexual health outcomes and to reduce undesirable events such as STIs and unwanted pregnancies.

### The operational implementation of HIV PrEP requires considering chronic follow-up of HIV-negative women as part of a comprehensive SRH care package

The a priori acceptability of PrEP appeared to be very high (more than 95% of FSWs declared interest in such tools in the PrEP-CI survey). Beyond the availability of PrEP, the entire follow-up of HIV-negative FSWs needs to be (re)designed. Indeed, as efficacy trials have shown, oral PrEP requires quarterly monitoring of users/participants, including renal monitoring and the systematic screening of STIs, since syndromic screening is not sufficient [28]. However, the current priorities of public policies in Côte d’Ivoire and international donors are the identification of new HIV-positive cases and their referral for HIV treatment. It is necessary to develop tools and a new organization of care that allows for the chronic follow-up of HIV-negative FSWs for the proper use of, and adherence to, PrEP.

### In the context of a high prevalence of hepatitis B, antiretroviral drugs (ARVs) to prevent HIV cannot be made available without making the same ARVs available to treat hepatitis B

The prevalence of hepatitis B virus (HBV) is high in Côte d’Ivoire; different studies showed an HBV prevalence situated between 9.4% [29] and 11.1% [30] in the 2010s. A prevalence of hepatitis B surface antigen (HbsAg) of 6.2% was also estimated in a cross-sectional study conducted among FSWs in Abidjan in 2014–2015 [31]. In addition, few FSWs are vaccinated (only 5.2% in the PrEP-CI survey according to FSW self-reports). However, there is currently no free hepatitis B treatment program in Côte d’Ivoire, except for HIV/HBV coinfected patients. Tenofovir-based antiretroviral treatments used for HIV PrEP can also treat hepatitis B. It would be unethical to provide a drug freely for prevention when the same drug is not available for treatment. The implementation of HIV PrEP is an opportunity to articulate HIV prevention and HBV prevention/care.

### To minimize the stigma associated with entry into care, the care of HIV-infected FSWs and the preventive follow-up of HIV-negative FSWs should not be dissociated

On the one hand, FSWs face many situations of stigmatization and judgmental attitudes from their neighborhood, their entourage, and from health professionals, which complicate their access to care [32–34]. This is probably amplified for HIV+ FSWs, knowing that people living with HIV also face many stigmatizing situations [35–37]. On the other hand, SRH requires the concern all FSWs, whether or not they are infected with HIV. For these reasons, also supported by NGO field workers’ feedback, it seems essential not to dissociate the follow-up of HIV-infected and HIV-negative FSWs. It may also help to improve HIV care among FSWs.

### Follow-up from outreach activities (at prostitution sites) to community clinics is not sufficient

The NGOs Aprosam and Espace Confiance both carry out outreach activities with peer educators who go to prostitution sites to conduct HIV prevention and rapid testing. Occasionally, they also use mobile clinics to offer an additional syndromic screening of STIs. However, access to fixed community clinics that follow up on these outreach activities remains limited; Espace Confiance estimates that only half of the FSWs who are newly diagnosed with HIV at prostitution sites access a community clinic for HIV care. The interviews conducted in the PrEP-CI study showed that few FSWs were seeking care after unprotected sexual intercourse. The majority were not aware of the availability of prevention tools such as HIV post-exposition prophylaxis or the morning-after pill, despite the information provided by peer educators. They tended to self-medicate rather than visit a healthcare provider. FSWs revealed a reluctance to go to community health centers, mainly because they wished to remain anonymous (for fear of stigmatization by the neighborhood) and because opening hours did not fit their work schedules. In 2011, a KAP (knowledge, attitudes, practices) survey conducted among FSWs in 18 cities in Côte d’Ivoire showed that 81% had heard about a community clinic, but only 60% had ever accessed a community clinic at least once [38]. This could be relevant for implementing mobile clinics that operate directly at prostitution sites, not only for punctual activities, but also for long-term follow-up.

### The high mobility of FSWs is an obstacle to continuity of care

According to PrEP-CI data, FSWs migrate seasonally (especially during the coffee and cocoa season in San Pedro, between September and December) or more occasionally (when sex work is done in a different city from their home city). Therefore, their work periods are variable, from some days in a week or month to several months a year.

## Methods/design

Based on prior evidence, the ANRS 12381 PRINCESSE project aims to implement and evaluate, in Côte d’Ivoire, a community-based comprehensive sexual and reproductive healthcare package, including HIV PrEP, STIs, and HBV management, targeting FSWs.

### Study setting

The PRINCESSE study is taking place in San Pedro and its surroundings, Côte d’Ivoire, which is a region with farming businesses (coffee and cocoa in urban zones, and palm oil and hevea in rural areas), thus leading to a high degree of labor migration among men. The harbor of San Pedro is one of the most important harbors in West Africa and the world’s largest in terms of cocoa bean exports.

The PRINCESSE study was developed in collaboration with the Aprosam community-based organization, which delivers HIV prevention and testing services directly at prostitution sites (outreach activities) and provides HIV and SRH care services to FSWs through a community clinic in San Pedro. In the PrEP-CI study, HIV incidence was estimated at 3.2% in 2016–2017 among FSWs reached by Aprosam in this region [3].

### Study design and objective

The ANRS 12381 PRINCESSE project is a single-arm interventional cohort of FSWs aiming to develop, document, and analyze a community-based healthcare package that combines testing and prevention tools, including PrEP, immediate HIV treatment, the management of HBV, and SRH. The recruitment of the PRINCESSE cohort (baseline participants) started on November 26th, 2019 and is ongoing, partly due to the pandemic of COVID-19; the cohort is planned to last at least 30 months. Recruitment of participants is made possible by the Aprosam organization’s networks of peer educators and their access to the population.

### Sample size and power calculation

In total, the PRINCESSE cohort aims to include 500 FSWs at prostitution sites. A pragmatic approach was taken to determine the sample size of the study, considering the absorption capacity of the mobile clinic. The mobile clinic is organized to return to the same site every two weeks. Operating five days a week, ten different sites are covered. In order to ensure sufficient consultation time for each FSW, our target was a maximum of ten consultations per trip. Since PRINCESSE follow-up consists of a quarterly visit on average, with the same site being visited five to six times per quarter, the target number of FSWs followed up periodically for a mobile clinic is 5 (visits on each site per quarter) × 10 sites × 10 consultations/visit = 500 FSWs per mobile clinic. Considering an HIV prevalence of around 20% [2], it was anticipated to recruit 100 (500 × 20%) HIV-positive FSWs and 400 (500 × 80%) HIV-negative FSWs.

Most outcomes listed in Table 3 (see below) are proportions. A minimum sample of *n* = 402 is required to estimate the 95% confidence interval of an expected proportion of 50% with a precision of ±5. For an expected proportion of 10% (or 90%), with a precision of ±3, a minimum sample of *n* = 414 is required. To have 80% power to detect with an alpha risk of 5% an evolution of ±10 points (e.g. from 50 to 60%) between two time points, a minimum sample of *n* = 408 is required.

### Eligibility criteria for the PRINCESSE cohort

The inclusion criteria are:

- Being a woman over 18 years of age
- Self-reporting as being a sex worker
- Wishing to enroll in a regular clinical follow-up
- Agreeing to participate in the study and signing the informed consent form
- Not already participating in another biomedical or behavioral study on HIV, viral hepatitis, or STIs
- Regardless of HIV status (infected or not)
- Whether or not the participant has already taken antiretrovirals
- Whether or not the participant is already followed by Aprosam

### Field implementation

**Fig. 1:**
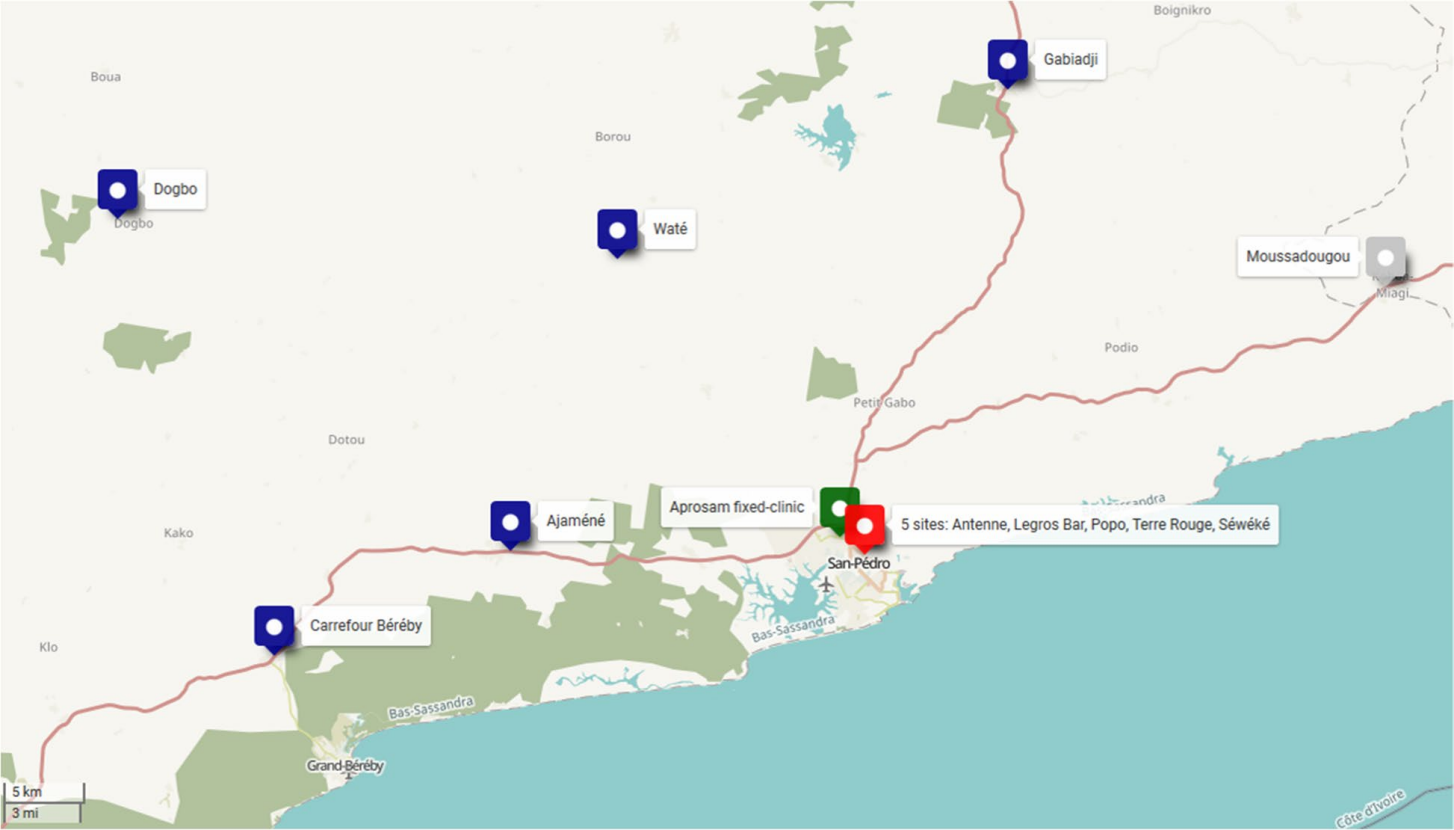
Implementation sites of the PRINCESSE project in the area of San Pedro, Côte d’Ivoire. Source: custom map by the authors using OpenStreetMap for background

The PRINCESSE intervention is delivered using a mobile clinic at 10 prostitution sites: 5 urban sites in the city of San Pedro and 5 rural sites around San Pedro (Fig. 1). Care services are also available at the fixed community clinic of Aprosam in San Pedro. Each prostitution site is visited every two weeks, and the operating hours vary according to each site.

After inclusion, participants are invited to be followed up with every 3 months, either in the mobile or fixed clinic, at their preference. FSWs can also access the clinics anytime they need to, in addition to planned quarterly visits.

The mobile clinic team comprises a medical doctor, a laboratory technician, a community counselor, a driver, two FSW peer-educators, and an independent interviewer.

### Healthcare offer

The PRINCESSE healthcare package is summarized in Table 1 and detailed below.

**Table 1:** Summary of PRINCESSE healthcare package per protocol visit

Time since inclusion (MO) W: week/M: month		M0	W2	M3	M6	M9	M12	M15	M18	M21	M24
**Initial screening** **(at inclusion)**	HIV testing	✓									
	HBsAg testing	✓									
**HIV prevention** **(if HIV-)**	HIV testing			✓	✓	✓	✓	✓	✓	✓	✓
	Creatinine *(if PrEP)*	✓		✓	✓	✓	✓	✓	✓	✓	✓
	HIV PrEP (TDF/FTC) *(if eligible)*		✓	✓	✓	✓	✓	✓	✓	✓	✓
	At PrEP interruption *(if AgHBs + or isolated HBcAb)*	*ALAT/ASAT (every 3 months) + HBV viral load if sign of hepatitis B reactivation*									
	HIV post-exposure prophylaxis	*when needed*									
**HIV care** **(if HIV+)**	Antiretroviral treatment	✓	✓	✓	✓	✓	✓	✓	✓	✓	✓
	CD4 and viral load (if HIV+)	✓			✓		✓		✓		✓
**HBV prevention (if HBsAg-)**	HBsAb testing	✓									
	HBcAb testing	✓									
	HBV vaccination *(if HBsAb- et HBcAb-)*	*3 × 1 doses if HIV- (M0, M1, M6), 4 × 2 doses if HIV+ (M0, M1, M2, M6)*									
**HBV care (if HBsAg+)**	HBeAg testing	✓									
	Creatinine *(if on TDF)*	✓		✓	✓	✓	✓	✓	✓	✓	✓
	ALT/AST/Platelets	✓			✓		✓		✓		✓
	Antiretroviral treatment (TDF/FTC) *(according to FIB-4 level and creatinine clearance level)*		✓	✓	✓	✓	✓	✓	✓	✓	✓
	HBV viral load	✓					✓				✓
**STI screening and care**	Syndromic STI screening	✓		✓	✓	✓	✓	✓	✓	✓	✓
	Systematic STI testing (Chlamydia PCR + Gonorrhea PCR + Syphilis rapid test)	✓					✓				✓
	Dysplasia testing (+ treatment when necessary)	✓					✓				✓
	STI treatment	*when needed*									
**Pregnancy screening and contraception**	Pregnancy test	✓		✓	✓	✓	✓	✓	✓	✓	✓
	Contraception (pills, injection or implant according to patient’s choice)	✓		✓	✓	✓	✓	✓	✓	✓	✓
	Emergency pill	*when needed*									
**Other**	Condom and lubricating gel	✓	✓	✓	✓	✓	✓	✓	✓	✓	✓
	Identification of addiction (tobacco, alcohol, drugs) and referencing when necessary			✓		✓		✓		✓	

#### Initial screening for HIV and hepatitis B at inclusion

All participants are screened for HIV during the inclusion visit using rapid tests according to the national testing algorithm: Determine® (sensitivity: 100%; specificity: 98.9%), Stat-pak® (sensitivity: 99.5%; specificity: 100%), and Biolane® in the case of a discrepancy. In the case of a new HIV diagnosis, a blood sample is collected for confirmation in a laboratory.

All participants are also screened for antigen HBs (AgHBs) using the rapid test Determine® (sensitivity: 93.6%; specificity: 100%) or VIKIA® (sensitivity: 96.5%: specificity: 99.9%). In case of a negative result for AgHBs, a blood sample is collected for anti-HBs and anti-HBc antibody testing in a laboratory. In case of a positive result for AgHBs, blood samples are collected for measuring HBV viral load, antigen HBe, platelets, ALT, and AST in a laboratory. The fibrosis level (F0-F1-F2 or F3-F4) is estimated using the FIB-4 indicator [39].

#### Antiretroviral treatment and hepatitis B vaccination

HIV treatment guidelines [40], HIV PrEP guidelines [41], and Hepatitis B guidelines [39] have been combined to produce the HIV/HBV care algorithm for the PRINCESSE participants (Table 2 and Fig. S1).

**Table 2:** Summary of HIV and HBV care algorithm for PRINCESSE participants

Group of patients	HIV and AgHBs negative	HIV-positive	HIV-negative AgHBs positive Fibrose ≥ F2 Elevated ALT and HBV DNA (eligible for HBV treatment)	HIV-negative AgHBs positive Fibrose < F2 Low HBV DNA et normal ALT (not eligible for HBV treatment)
**Hepatitis B vaccination**	If not immunized (anti-HBs and anti-HBc both negatives) 3 regular doses at M0, M1, M6	If not immunized (anti-HBs and anti-HBc both negatives) 4 double doses at M0, M1, M2, M6	–	–
**Antiretroviral prescription**	If creatinine clearance ≥ 60 mL/min and no contraindication TDF/FTC (1 pill/day) as oral PrEP	According to national guidelines As soon as possible, regardless of CD4 count Adapted regimen if HIV/HBV coinfection	If creatinine clearance ≥ 60 mL/min and no contraindication TDF/FTC (1 pill/day) as HBV treatment If creatinine clearance < 60 mL/min TDF with an adapted reduced dosage (1 pill every 1, 2, 3, or 7 days depending on the rate of creatinine clearance)	If creatinine clearance ≥ 60 mL/min and no contraindication TDF/FTC (1 pill/day) as oral PrEP

Participants who are HIV- and AgHBs-negative are offered the chance to initiate TDF/FTC (tenofovir and emtricitabine) for daily oral PrEP. For those interested, a blood sample is collected to measure creatinine clearance using the equation of Cockcroft & Gault. PrEP will only be prescribed if renal function is normal (creatinine clearance ≥60 mL/min); there is no contraindication to TDF and FTC; there are no clinical manifestations suggestive of primary HIV infection; and the patient is willing to take PrEP as prescribed and to enroll in required periodic medical follow-up [41]. Patients not immunized against HBV (HBs and HBc antibodies, testing negative for both) are offered the chance to be vaccinated (3 times a regular dose at M0, M1, and M6).

HIV-infected participants are followed according to Ivorian national treatment guidelines [40]. Antiretroviral treatment is proposed as soon as possible, regardless of CD4 count. In the case of HIV-HBV coinfection, antiretroviral treatment is adapted. Patients not immunized against HBV (HBs and HBc antibodies, testing negative for both) are offered the chance to get vaccinated (4 times a double dose, at M0, M1, M2, and M6).

For patients infected by HBV for whom treatment is recommended (a high level of HBV DNA or transaminases or fibrosis ≥F2), a blood sample is collected to measure creatinine clearance using the equation of Cockcroft & Gault. If creatinine clearance is ≥60 mL/min, TDF/FTC (1 pill/day) is prescribed. TDF/FTC is one of the regimens recommended by the WHO ([39], Table 6.1.a). For patients with creatinine clearance below 60 mL/min, only tenofovir (TDF) is prescribed at a reduced dosage, as recommended by the WHO ([39], Table 9.1): 50–59 mL/min: one 300 mg tablet every 24 h; 30–49 mL/min: one 300 mg tablet every 48 h; 10–29 mL/min: one 300 mg tablet every 72–96 h; and < 10 mL/min: one 300 mg tablet every 7 days. These patients are advised that there is no demonstrated prophylactic effect against HIV, with non-daily TDF, and will be encouraged to maintain the use of other HIV prevention tools.

For patients infected by HBV for whom treatment is not yet recommended (a low level of HBV DNA or ALT or fibrosis <F2), they are offered the chance to initiate TDF/FTC for oral HIV PrEP. For those interested, a blood sample is collected to measure creatinine clearance using the equation of Cockcroft & Gault. PrEP is only prescribed if renal function is normal (a creatinine clearance ≥60 mL/min); there is no contraindication to TDF and FTC; there are no clinical manifestations suggestive of primary HIV infection; and the patient is willing to take PrEP as prescribed and to enroll in the required periodic medical follow-up [41].

For all participants who are HIV-negative, HIV testing is proposed every 3 months, regardless of their PrEP status. In the case of HIV seroconversion, patients are given the chance to initiate an adapted antiretroviral regimen as soon as possible.

#### STI screening and care

Syndromic screening (clinical examination) is offered quarterly and at each non-protocol visit if needed. In the case of STI suspicion, national treatment kits are provided for free according to the national algorithm of the Ministry of Health.

This quarterly syndromic screening is completed annually through a biological screening (PCR chlamydia and gonococcus, rapid syphilis test, vaginal and anal swab) at M0, M12, and M24 because of the importance of asymptomatic STIs in women in general and among FSWs in particular. In the case of a positive rapid syphilis test, a blood sample is taken for a laboratory evaluation. Patients are called back by telephone for a complimentary visit if treatment is necessary based on the tests’ results.

Screening of condylomas and dysplastic lesions of the cervix is also proposed annually using acetic acid and Lugol. Treatment by thermoablation is available both in mobile and fixed clinics. If advanced lesions are diagnosed, conization and referral to a specialized medical service are proposed if necessary.

#### Pregnancy screening and contraception

Participants could also benefit from a reproductive health package, including free-of-charge non-barrier contraception (pill, injection, or implant; it is the participant’s choice), quarterly pregnancy tests, and emergency contraceptive pills when needed.

#### Other services

In addition, PRINCESSE healthcare includes menstrual management counseling with the offer of menstrual cups; a biannual identification of addiction (tobacco, alcohol, drugs), and referral to dedicated services when necessary; free condoms; and lubricating gel.

### Specific research objectives and principal outcomes/evaluation criteria

We defined six specific objectives to evaluate PRINCESSE intervention:

1. To analyze access to care and retention in care and, more generally, female participants’ healthcare trajectories through a quarterly follow-up of FSWs (infected with HIV or not)
2. To measure female participants’ health outcomes over time using clinical, behavioral, and social indicators
3. To assess PrEP initiation, use, and adherence
4. To compare HIV management in the PRINCESSE system with existing routine treatment and care
5. To measure HBV testing, vaccination, and treatment as part of decentralized management integrated with HIV PrEP, and possible interactions between HIV PrEP and HBV infection
6. To document the unexpected consequences (positive or negative) of the PRINCESSE system on the everyday lives of female participants in particular, and on the sex industry in general
7. To evaluate the impact of vaginal microbiota on bacterial sexually transmitted infections, human papillomavirus (HPV) infections, and associated cervical lesions; the impact of the HPV type distribution on the vaccinal strategy and the added value of HPV PCR for the primary screening of cervical cancer; and the impact of antimicrobial resistance on STI guidelines.

Due to the complexity of the intervention, identifying a unique main criterion over a multidimensional and multidisciplinary evaluation would be simplistic. For each of the six specific objectives, we defined a list of evaluation criteria that are neither exhaustive nor limiting (Table 3).

**Table 3:** Specific objectives and definitions of the main outcomes of the PRINCESSE project

Specific objectives	Principal outcomes	Population	Time Frame
*SO1. Access to care, retention, and healthcare pathways*	Completion rate of quarterly visits: proportion of completed study visits	All PRINCESSE participants	Up to 24 months
*SO2. Clinical, behavioral, and social evolutions*	Proportion with at least one diagnosed STI (chlamydia, gonococcus, or syphilis) detected by PCR or rapid test	All PRINCESSE participants	Up to 24 months
	Occurrence of an unwanted pregnancy in the last 12 months, orally reported	All PRINCESSE participants	Up to 24 months
*S03. Initiation, practices, and compliance with PrEP*	Initiation of PrEP: proportion having initiated PrEP	PRINCESSE participants eligible for PrEP	Over 24 months
	Adherence to PrEP: proportion being adherent (measured through self-report, pill count, and drug detection in plasma)	PRINCESSE participants on PrEP	Up to 24 months
*SO4. Comparison of HIV care (HIV+ patients of the PRINCESSE cohort* vs. *HIV+ patients of the NGO Aprosam not belonging to the PRINCESSE cohort)*	Number of participants in HIV care at 18 months (retention)	PRINCESSE participants who are HIV-infected at baseline + HIV-infected patients of Aprosam	18 months
	Occurrence of virological failure: proportion with two consecutive detectable viral loads	PRINCESSE participants who are HIV-infected and have initiated antiretroviral treatment + HIV-infected patients of Aprosam	Over 24 months (survival analysis)
*S05. Prevention and care of hepatitis B*	HBV vaccination rate: proportion with complete vaccination (3 doses if HIV-negative, 4 double doses if HIV-positive) at the end of the trial	PRINCESSE participants needing hepatitis B vaccination	Over 24 months
	Initiation and number of participants on TDF (retention) for patients with treatment for HBV mono-infection	PRINCESSE participants with a positive HBs-antigen and an F3-F4 fibrosis	Over 24 months
	Proportion with an increase in transaminase level (flares) after PrEP discontinuation	PRINCESSE participants who started and stopped PrEP and with a positive HBs-antigen	Within 12 months after PrEP discontinuation
*SO6. Unintended consequences of the PRINCESSE intervention*	Number of adverse social events occurring in participants’ daily lives	All PRINCESSE participants	Over 24 months
	Qualitative evaluation of undesired social events that occurred in the daily lives of participants and non-participants	PRINCESSE participants and non-participants FSWs in the targeted area of the intervention	Over 24 months
*SO7. Vaginal microbiota, HPV infection types, and antimicrobial STI resistance*	Proportion of cervical lesions at M0 and M12	All PRINCESSE participants	12 months
	Proportion of high-risk HPV infection included in quadrivalent and nonavalent vaccines at genital and anal levels at M0 and M12	All PRINCESSE participants	12 months
	Proportion of *M. genitalium*, N. gonorrheae, *C. trachomatis* infections at genital level at M0 and M12 Proportion of *M. genitallium* and N. gonorrheae antimicrobial resistances	All PRINCESSE participants	12 months

### Data collection

In the PRINCESSE cohort, four data points are collected:

(i) P1. Clinical and safety data: Dates and localization of medical consultations, the results of clinical examinations, clinical records of inclusions and follow-ups (including basic socio-behavioral indicators), adverse events, and prescriptions carried out as part of the PRINCESSE cohort.
(ii) P2. Socio-behavioral questionnaires: Sociodemographic characteristics, sexual practices and behaviors, HIV and HBV testing history, PrEP adherence/compliance, contraception, abortion, menstrual management, addictions, mental health, quality of life, perceived health, living standards, and social support.
  An independent interviewer is carrying out these questionnaires every six months during or around quarterly visits (M3, M9, M15, M21). There are no questionnaires collected at M0 to avoid the burden on inclusion visits. However, the main socio-behavioral indicators are collected at baseline in the clinical records of the included patients.
(iii) P3. Biological data: Results of lab tests performed on samples collected during medical consultations as part of the healthcare package; blood specimens and anal and vaginal swabs are also collected at M0, M12, and M24 to constitute a biobank.
(iv) P4. In-depth qualitative interviews with female participants: Semi-structured interviews are carried out with the participants of the PRINCESSE cohort by a scientist trained in qualitative methods.
  The interviews address several topics, such as participants’ personal experiences of the PRINCESSE project in general, how they appropriate the proposed tools, and more specifically, their understanding, daily experiences, and difficulties encountered with PrEP.

Four other additional waves of data collection, not included in the PRINCESSE cohort, are scheduled:

(i) A1. Capture the medical and activity records of Aprosam for PRINCESSE participants: Along with the PRINCESSE project, Aprosam continues its routine activities, including raising awareness, condom distribution, and outreach HIV testing conducted by peer educators. These routine activities, as well as medical consultations carried out within the community clinic of Aprosam, are recorded with specific identifiers. To complete the healthcare pathways of participants and their background with Aprosam, data routinely collected by Aprosam in medical records and registers of activities are gathered only for participants who gave specific consent.
(ii) A2. Capture of medical records of HIV+ FSW patients not participating in the PRINCESSE cohort and routinely examined by Aprosam: Beyond the HIV+ FSWs included in the PRINCESSE cohort, Aprosam proceeds with HIV care services already implemented for other infected FSWs within its community clinic based in San Pedro. To compare the usual HIV care of Aprosam’s clinic and the PRINCESSE cohort HIV care, comparative medical data are collected from medical records of Aprosam’s HIV+ patients, registered as FSWs and not participating in the PRINCESSE cohort. Retrospective data from the 24 months preceding implementation of the PRINCESSE cohort is also collected. Only indicators routinely documented as part of the usual activities of Aprosam are collected anonymously and encrypted.
(iii) A3. In-depth qualitative interviews with key informants in the FSW community: Semi-structured interviews are carried out within the community of FSWs by a qualitative scientist to complete interviews carried out with participants (P4). These interviews are carried out after obtaining informed consent to meet two objectives: (a) to document the reasons why some women refuse to participate in the PRINCESSE cohort by exploring, in particular, relationships between the sex work community and NGOs and social representations of healthcare and caregivers; (b) understand the impacts of PrEP in particular, and of the PRINCESSE cohort in general on the community of FSWs, beyond the participants.
(iv) A4. In-depth qualitative interviews with PRINCESSE follow-up actors (peer educators and caregivers): Semi-structured interviews are carried out with peer educators and caregivers involved in the PRINCESSE project by a qualitative scientist. These interviews aim to document how PRINCESSE follow-up actors appropriate the different tools, and describe the project implementation and eventual modifications from the planned program. Barriers and facilitators to retention in care for participants, and the positive or negative impacts of the PRINCESSE project on the sex work community, in general, are also documented through these interviews.

Each wave of data collection will help to answer one or several specific research objectives (Table 4).

**Table 4:** Data collection for each specific research objective

	Specific research objectives						
Data collection	SO1 Access to care, retention, and healthcare pathways	SO2 Clinical, behavioral, and social evolution	SO3 Initiation, practices, and compliance with PrEP	SO4 Comparison of HIV care (PRINCESSE vs. current)	SO5 Prevention and care of hepatitis B	SO6 Unintended consequences of the PRINCESSE intervention	SO7 Vaginal microbiota, HPV infection types, and antimicrobial STI resistance
*PRINCESSE cohort*							
**P1.** Clinical and safety data	X	X	X	X	X		X
**P2**. Socio-behavioral questionnaires	X	X	X		X	X	
**P3**. Biological data		X	X	X	X		X
**P4.** In-depth interviews with participants	X		X			X	
*Additional data collection*							
**A1.** Medical and activity records of Aprosam (PRINCESSE participants)	X						
**A2.** Medical records of HIV+ FSW patients (not participating in the PRINCESSE cohort)				X			
**A3.** In-depth interviews with members and key informants of the FSW community			X			X	
**A4**. In-depth interviews with PRINCESSE follow-up actors	X		X			X	

### Data management

Cohort data (P1, P2) are collected by the community counselor, the laboratory technician, and the medical doctor. All paper forms are returned to Aprosam’s headquarters and entered into an electronic clinical database that is managed and hosted by PAC-CI (a French and Ivorian research institute based in Abidjan).

Biological results are returned by the different laboratories to Aprosam or PAC-CI, and then entered into the clinical database using dedicated trial IDs.

Aprosam medical staff can access the clinical database through a secure portal. Each staff member has a secure individual account. The mobile clinic is equipped with computers and Internet access.

From the electronic clinical database, analytical datasets are generated by removing all direct identifiers and by using distinct IDs to avoid direct linkage of analytical datasets with paper forms. All analytical datasets have been declared to CNIL (French National Commission on Informatics and Liberty), and all data procedures are compliant with the European General Data Protection Regulation.

Data monitoring is done by Mereva, PAC-CI’s methodological centre.

## Discussion

Based on a pilot qualitative and quantitative survey and cobuilt with community NGOs [5], the PRINCESSE project for FSWs is one of the first interventions offering HIV oral PrEP as part of a more global sexual healthcare package, targeting both HIV-negative and HIV-positive women. A second innovation is the combination of HIV PrEP and viral hepatitis B care. For HIV-negative and HBV-negative women, PrEP is not mandatory to benefit from the other package components (e.g., STI screening and care, family planning). Finally, PRINCESSE is one of the very few programs implementing mobile clinics for chronic/quarterly care.

In terms of research, PRINCESSE is a comprehensive, interdisciplinary project that combines the clinical, biological, epidemiological, and social sciences. This interdisciplinarity is reflected in the diverse objectives and outcomes being investigated. Research questions are not limited to the participants and consider healthcare workers/implementing staff and the local sex work community.

Operational challenges are numerous and linked, among others, to the high mobility of participants through different prostitution sites and in different parts of the country, the motivation of healthcare workers throughout the entire project, the relationship with the sex work community and its perceptions of the program, the logistics of the mobile clinic (road conditions, truck breakdowns), the implementation of electronic medical records, or the adaptation of activities due to health measures linked to the COVID-19 pandemic. Documenting all these logistic and operational aspects allows for the gathering of crucial information for the project’s replication and transferability.

One component of the sexual healthcare package also represents a specific challenge: oral HIV PrEP has been implemented for FSWs in different African countries through several demonstration projects, and adherence to this tool has been relatively low [42–45]. The daily uptake of PrEP is not always adapted to FSWs’ life context. More broadly, the mobility of FSWs and the stigmatization they face from their neighborhood or healthcare professionals complicate their access to care and their retention in demonstration projects [20, 21, 46, 47]. Beyond the development of community support and mobile clinics to limit these difficulties, exploring the suitability of long-acting PrEP to this population could be useful [48].

Finally, the PRINCESSE project aims to understand whether the offer of all sexual healthcare directly at prostitution sites is sufficient to alleviate structural barriers in access to care.

## Supplementary Information

**Additional file 1: Figure S1**. HIV and HBV care  algorithm for PRINCESSE participants.

## Data Availability

The datasets generated during the current study are not publicly available due to their sensitivity (health data) and the fact that the reidentification of participants by cross-referencing data is possible. Access to pseudonymized analytical datasets is possible from the principal investigators (SE and JL) upon reasonable request and under a confidentiality and partnership agreement. For future publications, ad hoc, fully anonymized datasets will be generated and released into a public repository. At the end of the project, fully anonymized datasets (including precision loss) will be generated for long-term archiving.

## Abbreviations

CNESVS: Comité National d’Éthique des Sciences de la vie et de la santé de la Côte d’Ivoire
FSWs: Female sex workers
HBcAb: hepatitis B core antibody
HbsAg: hepatitis B surface antigen
HBV: hepatitis B virus
HPV: Human papillomavirus
NGO: Non-governmental organization
PrEP: Pre-exposure prophylaxis
SRH: Sexual and reproductive health
STIs: Sexually transmitted infections
TDF/FTC: Tenofovir and emtricitabine
