# Supplementary material for: A community-based healthcare package combining testing and prevention tools, including pre-exposure prophylaxis (PrEP), immediate HIV treatment, management of hepatitis B virus, and sexual and reproductive health (SRH), targeting female sex workers (FSWs) in Côte d’Ivoire: the ANRS 12381 PRINCESSE project
Source: BMC Public Health. 2021 Dec 4;21:2214. doi: 10.1186/s12889-021-12235-0 (PMC8642977; doi:10.1186/s12889-021-12235-0)
Supplement: Supplementary file 1 — Additional file 1: Figure S1. HIV and HBV care algorithm for PRINCESSE participants. [file 12889_2021_12235_MOESM1_ESM.pdf]

Supplementary Electronic Material

Figure S1. HIV and HBV care algorithm for PRINCESSE participants

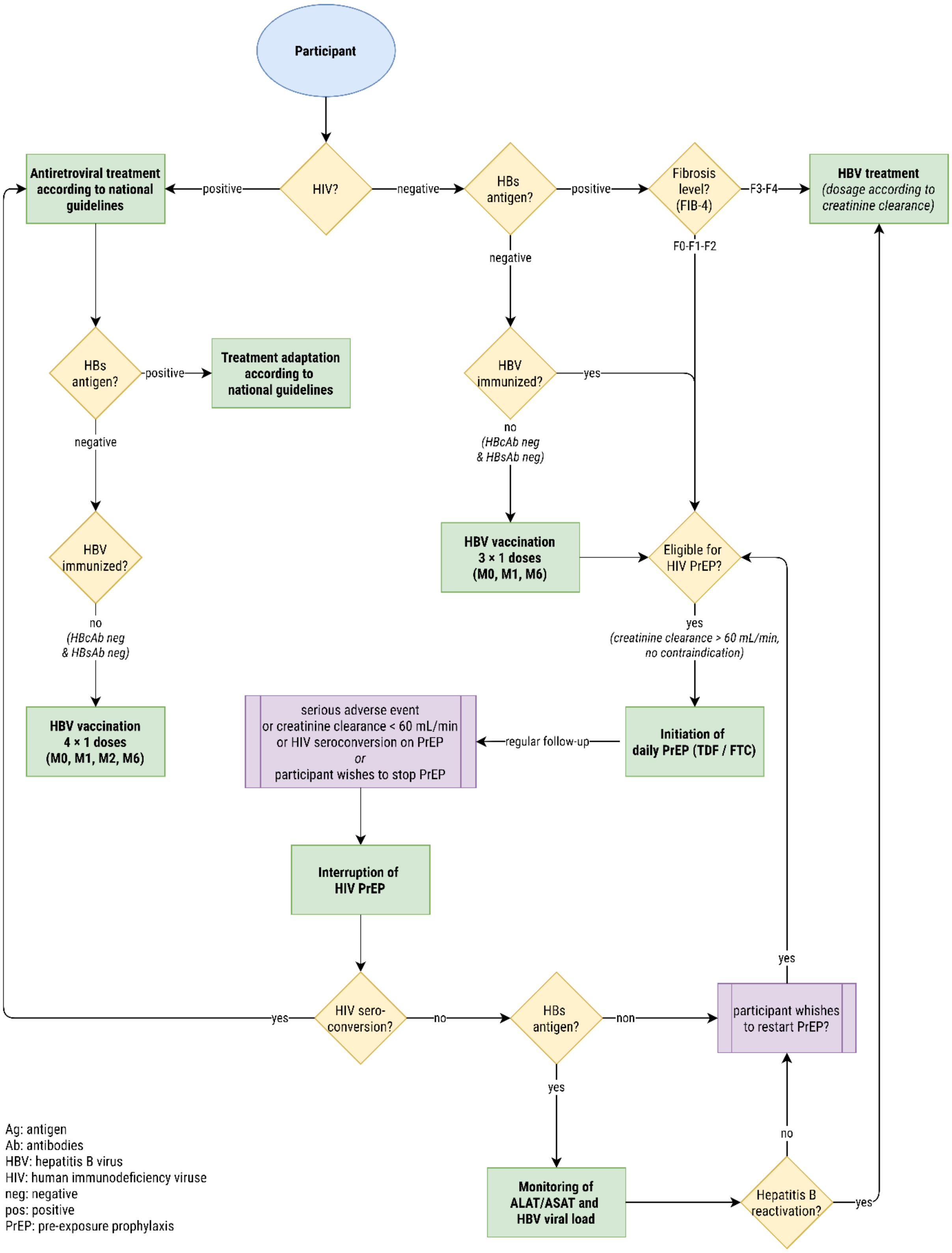

Ag: antigen
Ab: antibodies
HBV: hepatitis B virus
HIV: human immunodeficiency viruse
neg: negative
pos: positive
PrEP: pre-exposure prophylaxis

S2. PRINCESSE Study group

Institutions and authors are sorted alphabetically.

Investigators-Coordinators

PAC-CI, Côte d'Ivoire: S Eholié
Ceped, IRD, Université de Paris, Inserm, France: J Larmarange

Implementation team

Aprosam, San Pedro, Côte d'Ivoire: A Agoua (medical investigator), KP Anvo, S Akou, ML Brou, Cécé, Dodo, Flore, Francas, Kiki, P Kouassi, J Kouassi, Mama Kate, Merveille, Miss Falone, Nemy, Roky, Sly, C Zebago (medical investigator), GH Zonhoulou Dao (medical investigator)
CEDRES, Abidjan, Côte d'Ivoire: E Koné-Bravo
PAC-CI, Abidjan, Côte d'Ivoire: MN Nouaman (project coordinator)

Data management & monitoring

PAC-CI/MEREVA, Abidjan, Côte d'Ivoire: MN Nouaman, A Kouamé, S Lenaud, C Yao

Study Team

Aprosam, San Pedro, Côte d'Ivoire: A Agoua, C Zebago, GH Zonhoulou Dao
Bordeaux Population Health Research Center, Université de Bordeaux, Inserm, IRD, France: X Anglaret, M Plazy
Ceped, IRD, Université de Paris, Inserm, France: V Becquet, P Biligha, J Larmarange
CES, Université Paris 1 Panthéon-Sorbonne, France: C Pougue Biyong
Espace Confiance, Abidjan, Côte d'Ivoire: C Anoma
Harvard University, Boston, US: K Freedberg
Hôpital Saint-Antoine, Paris, France: A Boyd, K Lacombe
Ined, Paris, France: V Becquet
IPLESP, Sorbonne Université, Inserm, Paris, France: E Teyssou
PAC-CI, Abidjan, Côte d'Ivoire: X Anglaret, A Attia, P Coffie, C Danel, S Eholié, R Moh, MN Nouaman
SESSTIM, Université Aix-Marseille, Inserm, IRD, Marseille, France: M Fiorentino, B Spire
Sorbonne Université, Hôpital Pitié-Salpêtrière, Paris, France: V Calvez, A Jary, V Leducq, AG Marcelin
Université de Bordeaux, GH Pellegrin, CHU de Bordeaux, France: C Bébéar
Université de Paris, AP-HP, GH Saint-Louis-Lariboisière-Fernand Widal, France: B Berçot

Scientific Advisory Board

K Lacombe (chair, Hôpital Saint-Antoine, Paris), I Ahiba Bobo (National AIDS Programme, Abidjan), E Allah Kouadio (National Viral Hepatitis Programme, Abidjan), N de Castro (Hôpital Saint-Louis, Paris), A Horo (PAC-CI, Abidjan), C Laurent (IRD, Montpellier), J Tetty (Bletty, Abidjan), B Vuylsteke (Institut de Médecine Tropicale, Anvers), M Zannou (Université d'Abomey-Calavi, Cotonou)

Study sponsor representatives

ANRS | MIE, Paris, France: M Ben Mechlia, V Doré, N Mercier, A Montoyo, C Rekacewicz

Internship students

ISPED, Université de Bordeaux, France: N Badirou, E Moreno
